# GLUT1 Regulates the Tumor Immune Microenvironment and Promotes Tumor Metastasis in Pancreatic Adenocarcinoma via ncRNA-mediated Network

**DOI:** 10.7150/jca.72161

**Published:** 2022-05-13

**Authors:** Fengjiao Li, Chong He, Hanming Yao, Weiling Liang, Xijiu Ye, Jianmin Ruan, Lijun Lin, Jinmao Zou, Shurui Zhou, Yuzhou Huang, Yaqing Li, Shaojie Chen, Kaihong Huang, Guoda Lian, Shangxiang Chen

**Affiliations:** 1 Department of Gastroenterology, Sun Yat-sen Memorial Hospital, Sun Yat-sen University, Guangzhou 510120, China.; 2 Department of Gastrointestinal Surgery, Sun Yat-sen Memorial Hospital, Sun Yat-sen University, Guangzhou 510120, China.; 3 Guangdong Provincial Key Laboratory of Malignant Tumor Epigenetics and Gene Regulation, Sun Yat-sen Memorial Hospital, Sun Yat-sen University, Guangzhou 510120, China.; 4 Department of Anesthesiology, Sun Yat-sen Memorial Hospital, Sun Yat-sen University, Guangzhou 510120, China.

**Keywords:** Pancreatic adenocarcinoma, GLUT1, ncRNAs, immune infiltration, metastasis

## Abstract

Pancreatic adenocarcinoma (PAAD) is a digestive tumor with extremely high malignancy. Previous studies have reported that Glucose transporter 1 (GLUT1) contributes to the aggressive tumor progression in various cancer types and indicates an unfavorable prognosis. However, the function of GLUT1 in PAAD remains largely unclear. Through pan-cancer analysis of GLUT1 expression, GLUT1 expression was significantly higher in several cancer types including PAAD. Survival analysis based on the GLUT1 expression showed that GLUT1 could serve as a predictor of poor prognosis. We further predicted and screened the candidate non-coding RNAs (ncRNAs) upstream of the GLUT1 mRNA through correlation analysis, and found that the CASC19/miR-140-5p axis contributing to the regulation of GLUT1 expression. Our study suggested a link exists between GLUT1 expression and selected immunity-related indicators. Subsequent analysis revealed overexpression of GLUT1 in pancreatic cancer specimens and patients with highly expressed GLUT1 expression had worse prognosis. Based on the significantly different expression of GLUT1, the possibility that GLUT1 participated in tumor progression was identified. Using online public databases, genes co-expressed with GLUT1 were screened and enriched to metastasis-related pathways by enrichment analysis. Additionally, functional assays verified that GLUT1 could function in the metastatic process of PAAD cancer cells. Therefore, we proposed that GLUT1 might serve as a role in tumor immunity and tumor metastasis, and was expected to be a prognostic factor in PAAD.

## Introduction

Pancreatic adenocarcinoma (PAAD) is an extremely malignant tumor with insidious onset and rapid development, leading to untimely diagnosis and dismal survival 1. The special tumor microenvironment of PAAD contributes to cancer therapeutic resistance and accelerates the process of recurrence and metastasis 2. Up to date, surgical resection is the most predominant treatment for PAAD patients 3. However, patients often lose the opportunity to receive radical operation for the lack of specific and effective biomarkers. The majority of patients have been diagnosed as advanced stages at first visit 4-6. It is helpful to recover novel predictors for diagnosis and treatment, which may serve as effective therapeutic targets for PAAD.

PAAD has a distinctive tumor microenvironment characterized by hypoxia 7 and apparent mesenchymal response 8. However, PAAD cells undergo metabolic reprogramming 9 and increased utilization of glucose 10 when facing vast energy and oxygen consumption 11. The glucose uptake process is mediated by glucose transporter proteins 12, particularly glucose transporter 1 (GLUT1), which function predominantly in metabolic activation 13 and the glycolytic process 14. GLUT1 distributes widely in human body, its expression tends to be low in normal tissues or benign lesions compared with corresponding tumor tissues, which partially indicates a rapid development and poor prognosis 15. Increasing evidences suggested that GLUT1 expression positively correlated with survival time and worse response to treatment 16-19. But the mechanisms remain unclear in PAAD, especially the regulatory network of GLUT1 and competitive endogenous RNAs (ceRNAs), as well as the relationship between GLUT1 and malignant biological behaviors of PAAD.

In total, pan-cancer analysis was carried out for in-depth research about the GLUT1 expression and survival analysis of patients who suffered from different human cancer types. We further predicted and screened the possible upstream regulatory non-coding RNAs (ncRNAs) of GLUT1 mRNA, microRNAs (miRNAs) and long non-coding RNAs (lncRNAs). To further explore the relevance of GLUT1 expression w tumor immunity, we performed correlation analysis on the gene expression level of GLUT1 and immunity-related indicators. Ultimately, we verified the results in clinical specimens and cancer cell lines. The following experiments further explored the function of GLUT1 and supported the notion that GLUT1 participated in promoting aggressive tumor behaviors.

## Materials and methods

### GEPIA database analysis

Gene expression profiling interactive analysis (GEPIA) online database primarily facilitated us to perform pan-cancer analysis and survival analysis of target genes based on The Cancer Genome Atlas (TCGA) and The Genotype-Tissue Expression (GTEx) databases 20. For pan-cancer analysis, we compared GLUT1 expression in tumor samples of 31 cancer types from the TCGA database and the corresponding normal samples from TCGA and GTEx databases. The results were shown in the form of dot plots and box plots. For survival analysis, Kaplan-Meier survival curves of overall survival (OS) and disease-free survival (DFS) assisted in evaluating the prognostic value of GLUT1 and candidate lncRNAs, Log-rank test was carried out to evaluate the survival differences. The expression relevance between GLUT1 and immune checkpoints (PD-1, PD-L1, and CTLA-4) was performed using Spearman's correlation coefficient.

### StarBase database analysis

StarBase database (version 2.0) was used to predict the candidate miRNAs of GLUT1 mRNA and the upstream lncRNAs of the selected miRNA 21. After screening, we detected the expression of selected miRNA or lncRNA and performed a survival analysis of miRNAs in PAAD. The intrinsic interactions between ncRNAs were also evaluated to identify the upstream miRNA and lncRNA of GLUT1 mRNA, including miRNA-mRNA interaction and miRNA-lncRNA interaction.

### Prediction of miRNAs

Upstream miRNAs for GLUT1 mRNA were predicted by the StarBase database, which consisted of several target-predicting programs (TargetScan 22, DIANA-microT 23, miRmap PITA, RNA22, miRanda, and PicTar 21). The miRNAs were included for the next analysis when appeared in at least two predicting programs. The relationships between miRNAs and GLUT1 were assessed by the StarBase database to identify the candidate miRNAs. MiRNAs that represented a negative correlation with GLUT1 expression and simultaneously exhibited down-regulation of expression in PAAD were selected as candidate miRNAs.

### Prediction of lncRNAs

Upstream lncRNAs for miR-140-5p were identified by the StarBase database and miRNet 2.0^24^. The relationships between lncRNAs and miR-140-5p were assessed by the StarBase database to identify the candidate lncRNAs. Criteria for selecting candidate lncRNAs were as follows: lncRNAs represented a negative correlation with miR-140-5p and a positive correlation with GLUT1 mRNA in PAAD.

### TIMER database analysis

Tumor immune estimation resource (TIMER) online website 25 was used for evaluating the relationship between different copy number variations of GLUT1 (arm-level deletion, diploid/normal, and arm-level gain) with immune cell populations (B cell, CD4+ T cell, CD8+ T cell, macrophage, neutrophil, and dendritic cell) in PAAD. Expression correlation analyses were carried out to determine whether there exists a relationship between GLUT1 and immune cell markers in PAAD. The expression correlation between GLUT1 and immune checkpoints (PD-1, PD-L1, and CTLA-4) was assessed by Spearman's correlation coefficient and adjusted for tumor purity. Results displayed in the form of scatterplots and significant differences were defined as *P* value < 0.05 (* *P* < 0.05, ** *P* < 0.01, *** P < 0.001).

### GSCA database analysis

Gene set cancer analysis (GSCA) online website^26^ was used for comparing GLUT1 expression levels with the infiltration levels of immune cells (B cell, CD4+ T cell, CD8+ T cell, macrophage, neutrophil, and dendritic cell) in PAAD respectively.

### LinkedOmics database analysis

LinkedOmics online website 27 was used for screening the genes which co-expressed with GLUT1 in PAAD. The co-expression analysis was measured by Pearson's correlation coefficient and visualized using scatter plots and heatmaps.

### Gene set enrichment analysis

Gene ontology (GO) functional and Kyoto Encyclopedia of Genes and Genomes (KEGG) pathway enrichment analysis for differentially co-expressed genes of GLUT1 were carried out using gene set enrichment analysis (GSEA). Genes co-expressed with GLUT1 were obtained from the LinkedOmics database for further analysis. The minimum and maximum gene set sizes were set to 5 and 5000, respectively.

### STRING analysis

STRING online website 28 was used for establishing protein-protein interaction (PPI) network centered on GLUT1 for Homo Sapiens.

### Tissue specimens and clinical information

All tissue specimens were collected from Sun Yat-sen University, Guangzhou, China. The inclusion criteria for patients were as follows: 1) pathologically confirmed as pancreatic adenocarcinoma, 2) underwent surgical resection, 3) patients with complete and accessible clinical information. Exclusive criteria were as follows: 1) complicated with other malignant or major diseases, 2) received neoadjuvant chemotherapy or radiotherapy before surgical resection. From January 2013 to December 2019, 67 specimens were included and clinical information of the corresponding patients was obtained for a follow-up study.

### Immunohistochemistry staining analysis

Paraffin-embedded pancreatic adenocarcinoma tissue sections were processed according to the following steps: deparaffinization, hydration, antigen retrieval, and blocking with goat serum. The sections were incubated with anti-GLUT1 antibody (1:1000) at 4℃ overnight and incubated with species-specific secondary antibody at 37℃ for 30 min. The sections were stained with DAB solution and hematoxylin sequentially. Representative images of immunohistochemistry (IHC) staining were acquired under the optical microscope.

### Cell culture and transfection

The human pancreatic adenocarcinoma cell lines (PANC-1 cell line and BxPC-3 cell line) were selected for subsequent experiments and cultured in DMEM medium with 10% fetal bovine serum at 37℃ in a humidified incubator with 5% CO2. PANC-1 cells or BxPC-3 cells were seeded in 12-well plates before transfection. Small interfering RNAs (siRNAs) were transiently transfected into cells with Lipofectamine 3000 at the final concentrations of 50 nM and the transfection efficiency of GLUT1 siRNA was assessed by Western blot analysis. Cells without transfection were treated as a control.

### Transwell migration and invasion assays

Cell migration and invasion abilities were quantified by the number of cells that penetrated through transwell chambers with polycarbonate filters of 8 μm pore size. For migration assay, PANC-1 cells or BxPC-3 cells were resuspended with serum-free DMEM medium (2×10^5^ cells/200 μL) and planted in 24‐well transwell chambers (the upper chamber). 500 μL DMEM medium with 10% FBS was added to the 24-well plates (the lower chamber). For invasion assay, chambers were pre-coated with 70 μL Matrigel to the upper filter and the remaining steps were performed as previously described. The cells on the Transwell membrane were fixed with methanol and stained with crystal violet.

### Scratch Assay

PANC-1 or BxPC-3 cells were seeded in 6-well plates which were pre-marked with horizontal lines across the well at even intervals. Straight lines were drawn perpendicular to the marked lines to make a scratch using sterile gun heads. The cells were incubated with serum-free DMEM medium. The scratch regions were photographed at 0 h and 24 h, respectively. The scratch-healing area was calculated by the following formula: Percentage of scratch-healing area = (0 h scratch area-24 h scratch area) / 0 h scratch area ×100%.

### Western blot analysis

Protein samples were separated and transferred to the polyvinylidene difluoride (PVDF) membrane for the subsequent steps. After blocking with 5% BSA, the PVDF membrane was incubated overnight with primary anti-GLUT1 antibody (1:1000) at 4 °C and then incubated for 2 h with HRP-labeled secondary antibody (1:5000) at room temperature. ECL was used to expose the image. β-actin was selected as an internal reference.

### Statistical analysis and graphing

Analyses were performed by web-based bioinformatics and the statistics were calculated by online databases. SPSS software (version 21.0) was used for statistical analysis and GraphPad Prism (version 8.0) was used for plotting graphs. The graphical abstract was drawn by online platforms, including the Figdraw tool and the Sangerbox tool. Statistical significance was defined as *P* value < 0.05 or Log-rank *P* value < 0.05 (* *P* < 0.05, ** *P* < 0.01, *** P < 0.001).

## Results

### GLUT1 expression in the pan-cancer analysis

**Figure 1:**
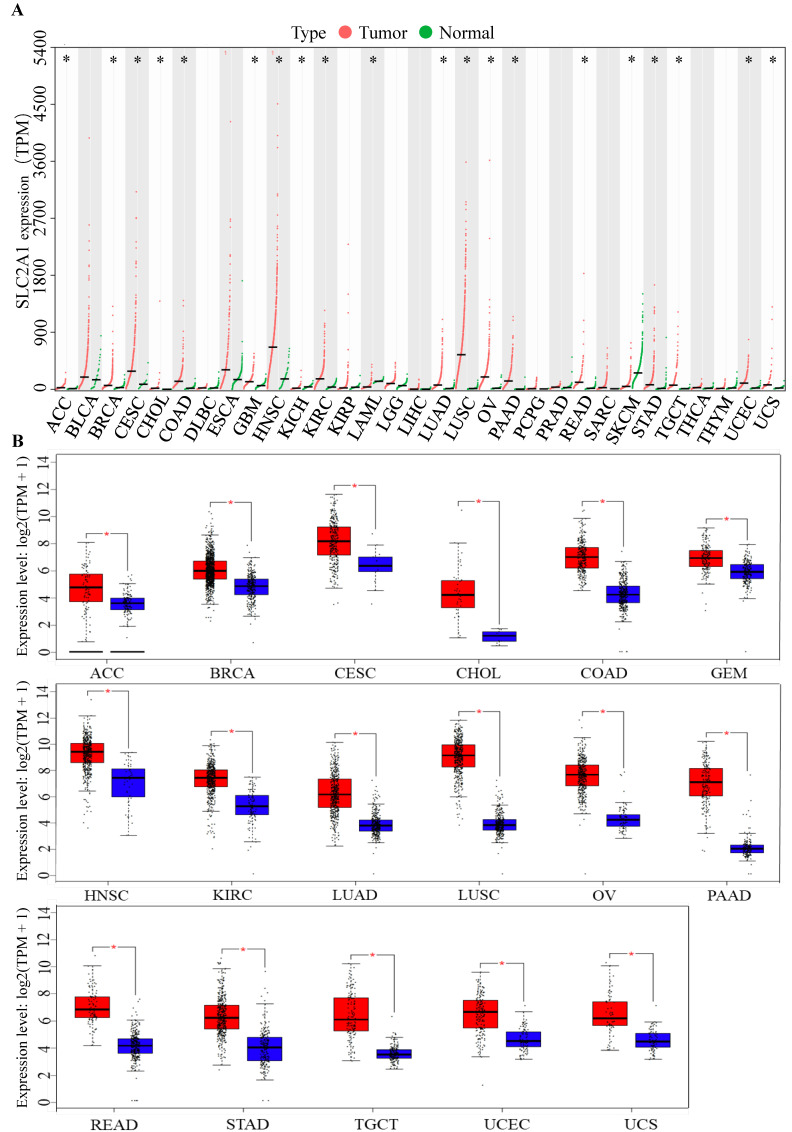
(A) Pan-cancer analysis of GLUT1 (TPM). (B) Pan-cancer analysis of GLUT1 expression presented in the form of box plots separately for each cancer type. (* P < 0.05; n.s, *P* > 0.05.)

To identify the role of GLUT1 in tumor progression, we detected GLUT1 expression levels in various cancer types. Pan-cancer analysis of GLUT1 was based on TCGA and GTEx databases, in which the tumor samples could match with control normal samples from TCGA and GTEx expression data from the same tissue. As illustrated in Figure 1A, a total of 31 types of human cancers were included and 20 cancer types exhibited significant GLUT1 expression differences. Among these cancers listed in the figure, we observed a significantly high expression level of GLUT1 in 17 cancer types (including ACC, BRCA, CESC, CHOL, COAD, GBM, HNSC, KIRC, LUAD, LUSC, OV, PAAD, READ, STAD, TGCT, UCEC, and UCS), and a significantly low expression level of GLUT1 in 3 cancer types (including KICH, LAML, and SKCM). To better understand gene expression changes, we separately investigated the specific expression levels of GLUT1 in the above 17 cancer types using the GEPIA database (Figure 1B).

### Survival analysis of GLUT1

**Figure 2:**
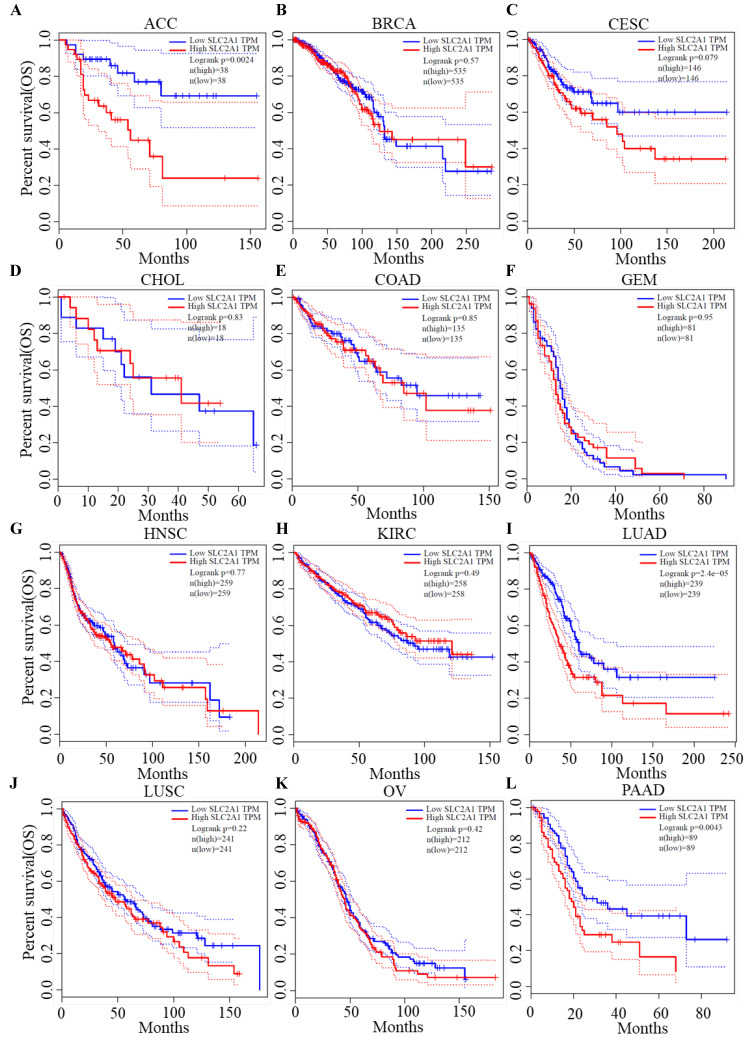

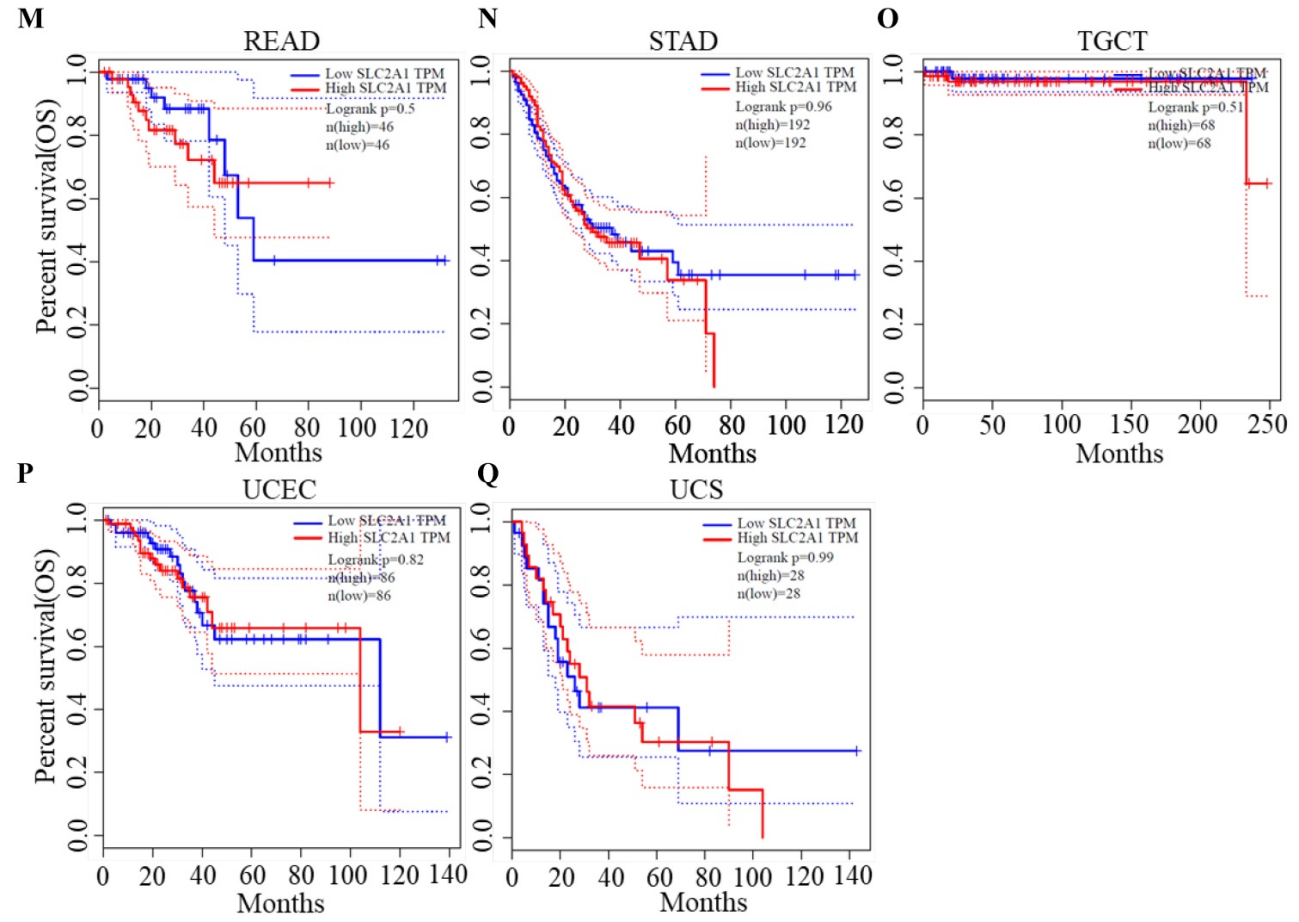
Kaplan-Meier survival curve for OS according to GLUT1 expression levels.

**Figure 3:**
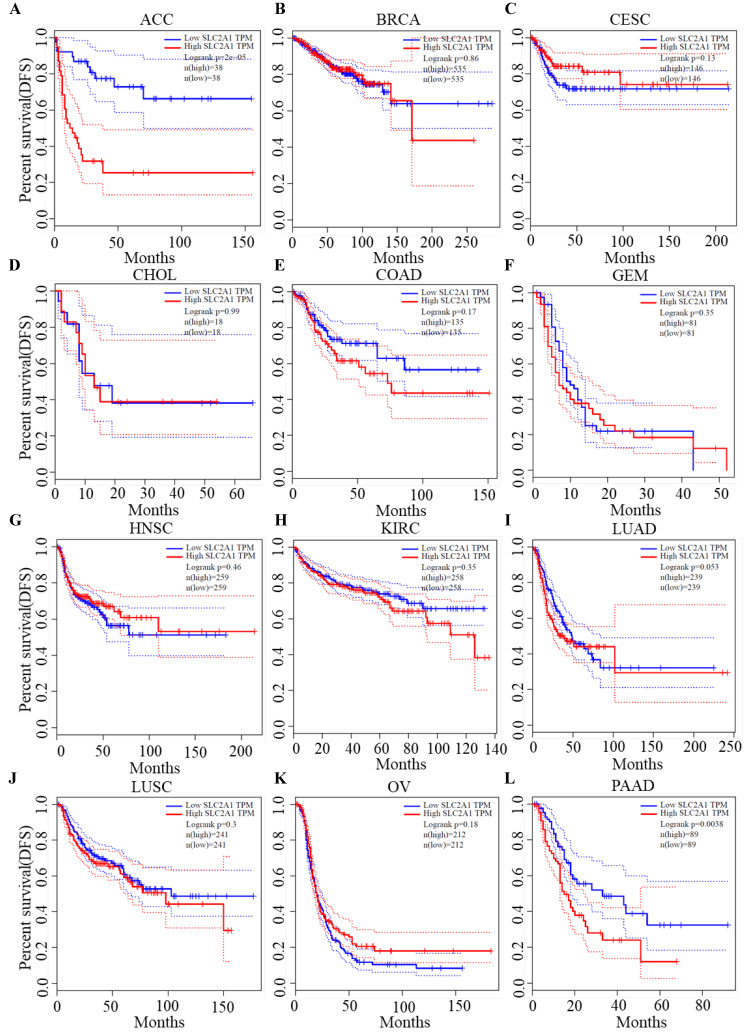

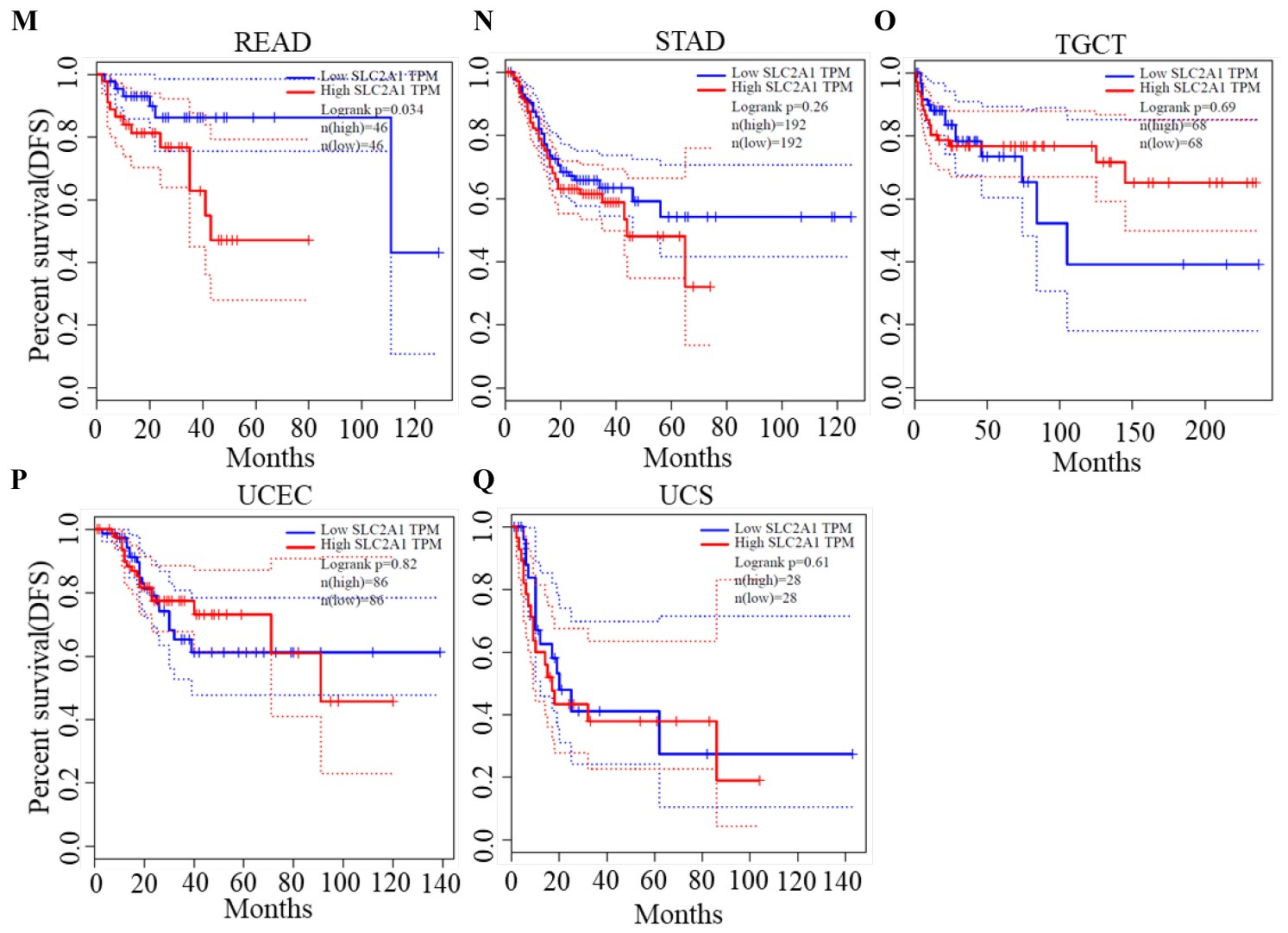
Kaplan-Meier survival curve for DFS according to GLUT1 expression levels.

We plotted Kaplan-Meier curves of OS and DFS in cancer types which presented upregulated expression of GLUT1. For the analysis of OS, GLUT1 expression level had a significantly negative association with OS in patients with ACC (Log-rank *P* value = 2.40e-03), LUAD (Log-rank *P* value=2.40e-05), and PAAD (Log-rank *P* value = 4.30e-03) (Figure 2). For the analysis of DFS, GLUT1 expression level had a significantly negative association with DFS in patients with ACC (Log-rank *P* value=2.00e-05) or PAAD (Log-rank *P* value=3.80e-03) (Figure 3). There were no statistically significant differences in OS or DFS depending on GLUT1 expression in cancers other than ACC and PAAD. The above results suggested that high GLUT1 expression might indicate worse outcomes in patients with PAAD.

### Prediction of the upstream miRNAs interacting with GLUT1 mRNA in PAAD

**Figure 4:**
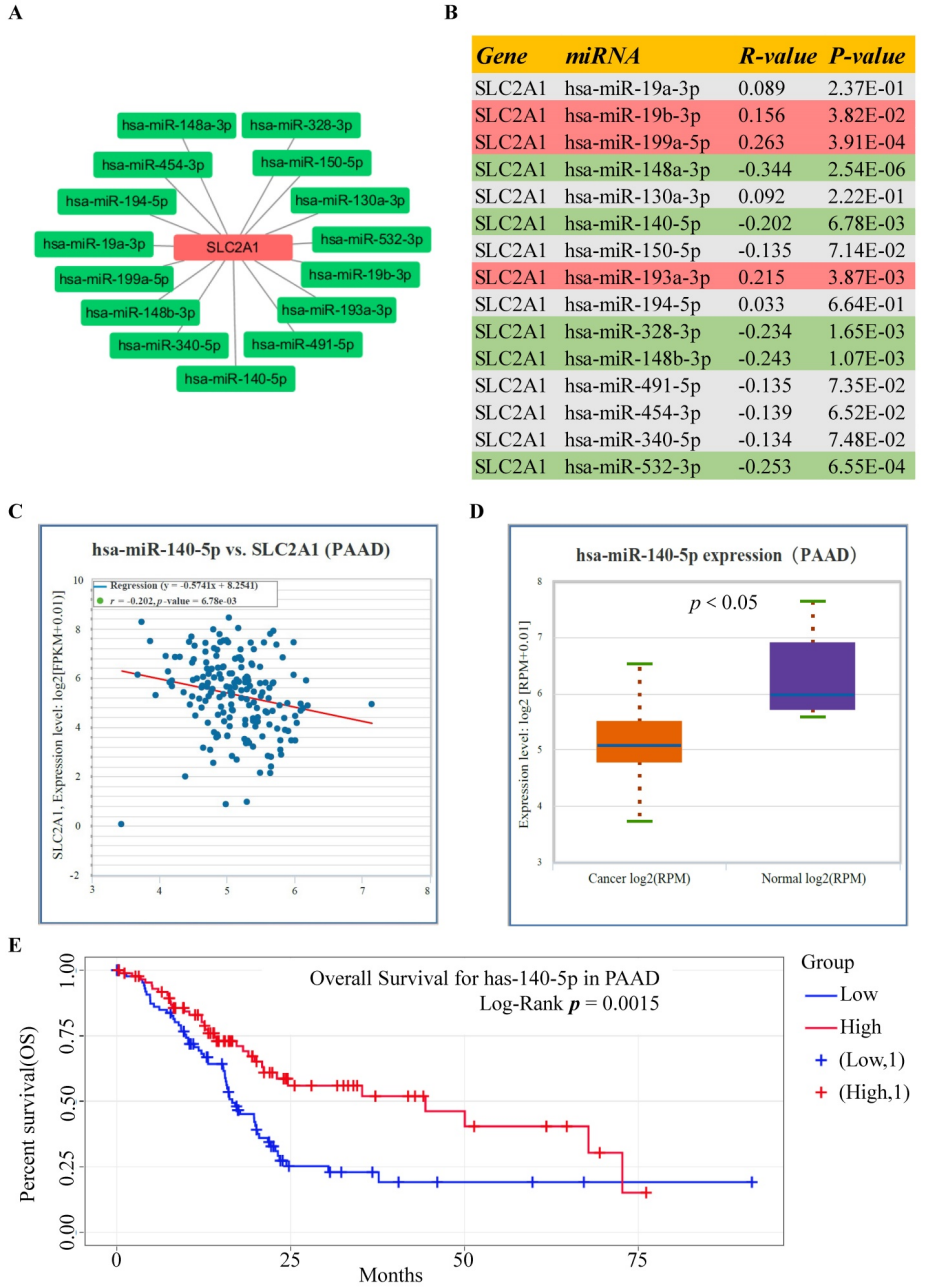
Screening upstream miRNAs interacting with GLUT1 mRNA in PAAD. (A) Candidate miRNAs interacting with GLUT1 mRNA visualized by Cytoscape software. (B) Correlation analysis between GLUT1 expression and candidate miRNAs in PAAD. (C) Correlation analysis between miR-140-5p and GLUT1 in PAAD. (E) miR-140-5p expression level in PAAD (D). The survival analysis of miR-140-5p in PAAD.

Using the StarBase database, the potential upstream miRNAs of GLUT1 mRNA were predicted and finally, 15 miRNAs were identified for further validation. Cytoscape software was performed to visualize candidate miRNAs, as well as indicated the interaction of the regulatory network (Figure 4A). All the predicted miRNAs which showed significant negative correlations with the corresponding mRNA were considered to be the potential upstream miRNAs of GLUT1. As listed in Figure 4B, miRNA-mRNA association analysis revealed that miR-140-5p, miR-148a-3p, miR-148b-3p, miR-328-3p and miR-532-3p were negatively related to GLUT1 mRNA, and miR-19b-3p, miR-193a-3p and miR-199a-5p were positively related to GLUT1 mRNA in PAAD. Figures 4C and 4D showed only miR-140-5p to be negatively correlated with GLUT1 mRNA and to be downregulated in PAAD. We further analyzed the prognostic value of miR-140-5p based on the StarBase database. The results of the survival analysis found that low miR-140-5p expression might indicate a worse prognosis (Log-rank *P* value=1.50e-03) (Figure 4E). These findings suggested that miR-140-5p could function upstream of GLUT1 mRNA in PAAD.

### Prediction of the upstream lncRNAs interacting with miR-140-5p in PAAD

**Figure 5:**
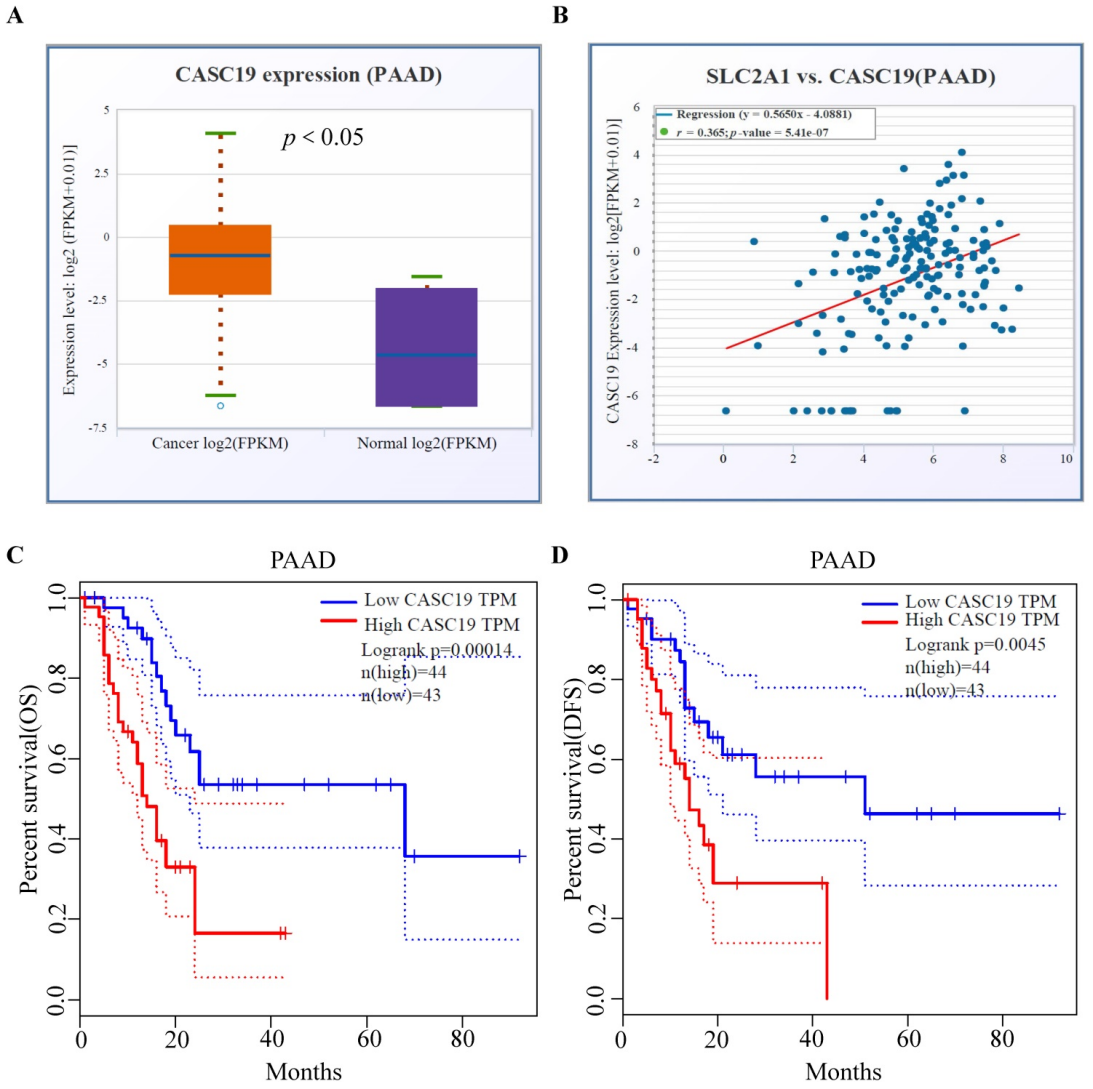
Prediction and identification of the upstream lncRNAs interacting with miR-140-5p in PAAD. (A) The correlation between CASC19 and GLUT1 mRNA in PAAD. (B) The CASC19 expression in PAAD. (C) Kaplan-Meier OS curve based on CASC19 expression level in PAAD using GEPIA database. (D) Kaplan-Meier DFS curve based on CASC19 expression level in PAAD using GEPIA database.

The upstream lncRNAs of miR-140-5p were screened through online tools. We found 37 candidate lncRNAs and utilized the DIANA tool to identify the interactions between predicted lncRNAs and miRNA-140-5p, in which, 20 lncRNAs had strong associations with miR-140-5p and showed significant positive correlation with GLUT1 mRNA (Table 1). As illustrated in Figure 5A, we analyzed the candidate lncRNAs expression in PAAD, and only CASC19 significantly upregulated in PAAD. Figure 5B showed the correlation analysis of GLUT1 mRNA and CASC19 mRNA in PAAD. Our next step was to assess the value of CASC19 through survival analysis. The CASC19 expression level was significantly negatively associated with OS (Log-rank *P* value=1.40e-04) and DFS (Log-rank *P* value=4.50e-03) for patients with PAAD (Figure 5C-D). According to the results based on the above predictions, only CASC19 could be a potential upstream lncRNA of miR-140-5p and GLUT1 mRNA in PAAD.

### Immune infiltration status of different GLUT1 expression levels in PAAD

**Figure 6:**
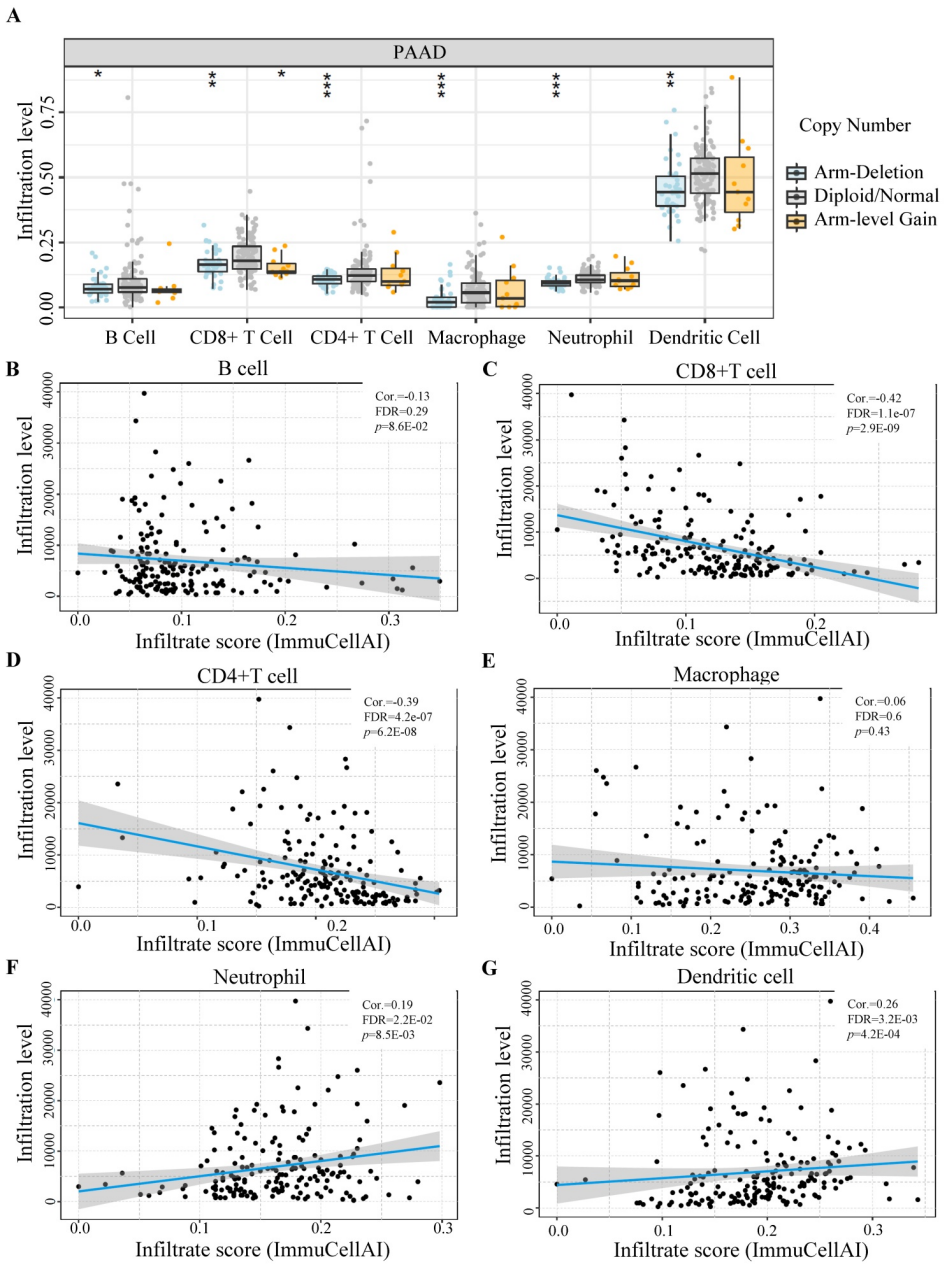
The influence of GLUT1 expression on immune cell infiltration in PAAD. (A) The copy number variation of GLUT1 acted on immune cell infiltration level in PAAD. (B-G) Correlation analysis between GLUT1 expression and different immune cell infiltration levels in PAAD.

Utilizing TIMER databases, we investigated the relationship between GLUT1 copy number variations and the levels of immune cell infiltration in PAAD. As shown in Figure 6A, the copy number alterations for SLC2A1 (encoding GLUT1) were classified into three states (including arm-deletion, normal, arm-level gain), and the percentage of infiltrating immune cells was represented on the longitudinal axis. The results in the figure indicated that copy number alterations of SLC2A1 could influence the immune cell infiltration levels (B cells, CD8+ T cells, CD4+T cells, macrophages, neutrophils, and dendritic cells) in PAAD. We also observed a significant negative relationship between GLUT1 expression and the infiltration levels of CD8+ T cells and CD4+T cells, as well as a significant positive relationship between GLUT1 expression and the infiltration levels of neutrophil and dendritic cells (Figures 6B-6G).

### Relationship between immune biomarkers and GLUT1 expression in PAAD

We detected the association between GLUT1 expression and biomarkers of immune cells through GEPIA and TIMER databases (Table 2). The GLUT1 expression was significantly linked to the biomarkers of B cell (CD20, CD70, and CD79A), CD8+ T cell (CD8A and CD8B), macrophage (CD68), M1 macrophage (COX2), neutrophil (CD55 and CCR7), and dendritic cell (HLA-DPB1, HLA-DRA, HLA-DPA1, and CD1C) in PAAD.

### Relationship between immune checkpoints and GLUT1 expression in PAAD

**Figure 7:**
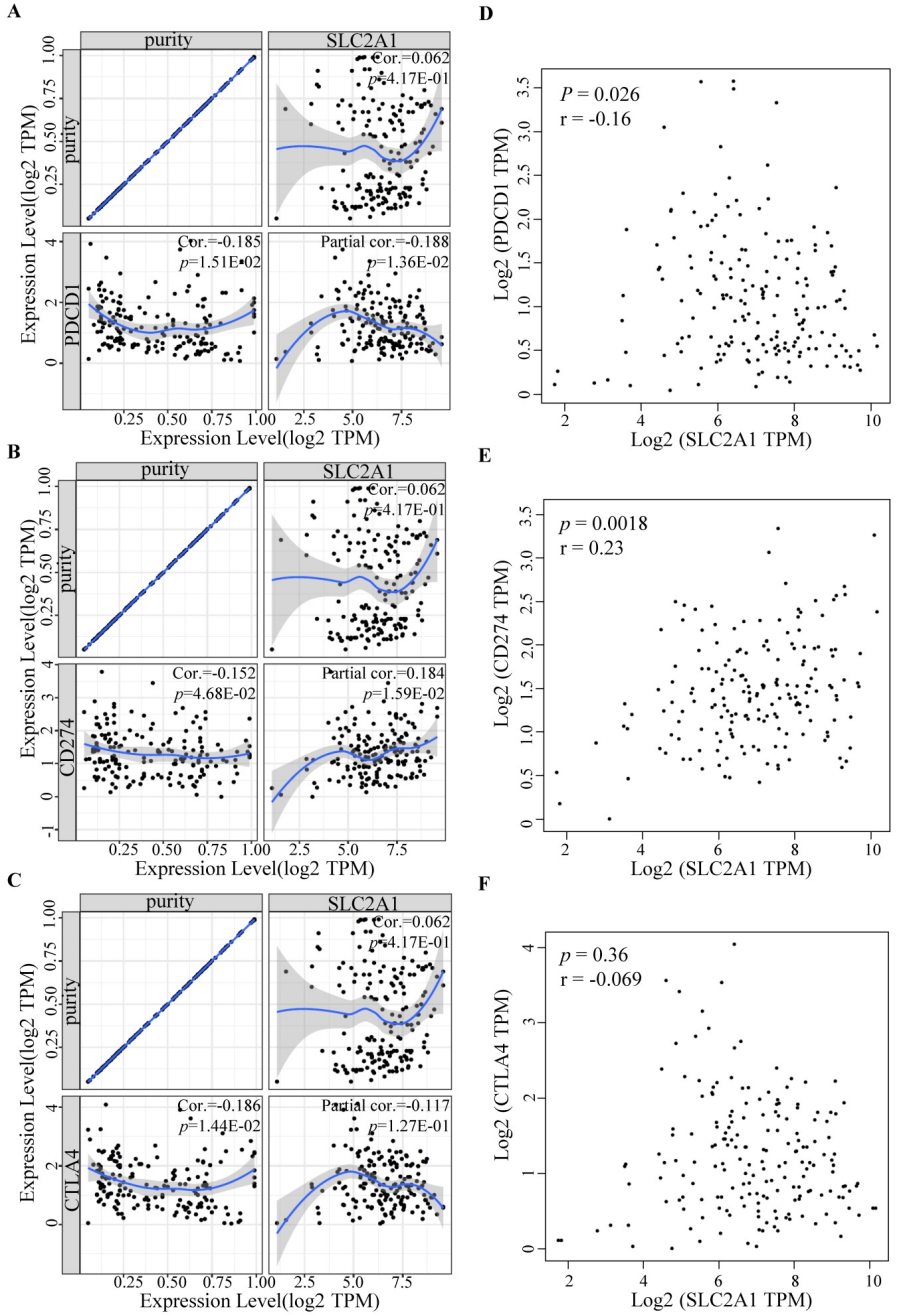
Relationship between immune checkpoints and GLUT1 expression in PAAD. (A) Relationship between GLUT1 expression and PD-1 expression adjusted by tumor purity in PAAD. (B) Relationship between GLUT1 expression and PD-L1 expression adjusted by tumor purity in PAAD. (C) Relationship between GLUT1 expression and CTLA-4 expression adjusted by tumor purity in PAAD. (D) The expression scatterplot of GLUT1 expression with PD-1 expression in PAAD. (E) The expression scatterplot of GLUT1 expression with PD-L1 expression in PAAD. (F) The expression scatterplot of GLUT1 expression with CTLA-4 expression in PAAD.

To further determine GLUT1 function in immune escape, we identified the expression levels of common immune checkpoints, including PD-1 (PDCD1), PD-L1 (CD274), or CTLA-4. Results revealed that GLUT1 expression positively correlated with PD-L1 (*P* value=1.59e-02) and negatively correlated with PD-1 (*P* value=1.36e-02) in PAAD, and no significance was observed between GLUT1 expression and CTLA-4 expression after adjustment for tumor purity (Figure 7A-C). The scatterplots were performed using the TCGA database to visualize the expression correlation between GLUT1 and immune-related checkpoints (PD-1, PD-L1, and CTLA-4), and the results revealed a similar variation tendency (Figure 7D-F).

### Association between GLUT1 expression and clinical characteristics of patients with PAAD

Given the substantially higher expression of GLUT1 in tumor samples, there might exist a possible link between GLUT1 expression and tumor progression. Consequently, we collected and analyzed the clinicopathological data of patients with PAAD from Sun Yat-sen Memorial Hospital inpatient databases to explore the possible mechanism of being a potential prognostic biomarker in PAAD. The clinical characteristics were listed in Table 3. We analyzed the data using the Chi-square test. The results demonstrated that high expression of GLUT1 was remarkably relevant to tissue size (*P* value=3.60e-02), the degree of tumor differentiation (*P* value=4.90e-02), and lymph node metastasis (*P* value=1.80e-02).

### Gene set enrichment analysis of genes co-expressed with GLUT1 in PAAD

**Figure 8:**
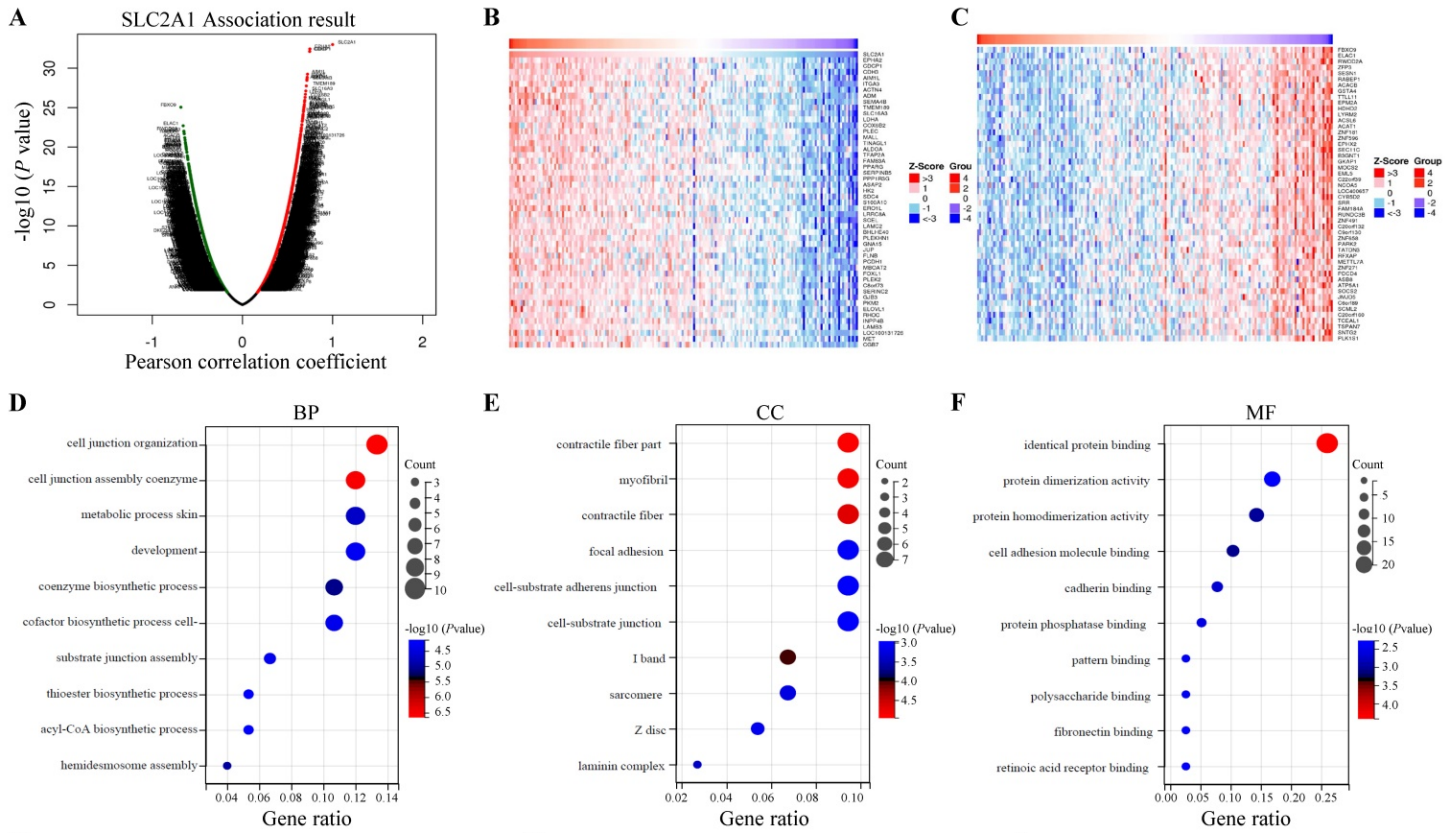

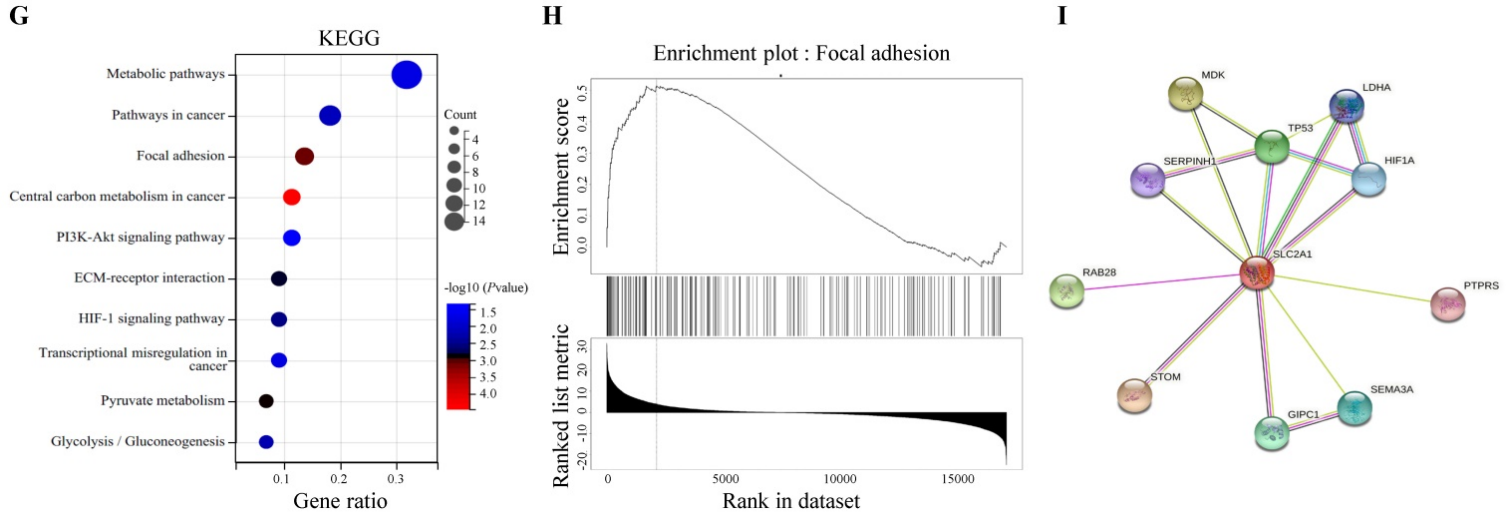
Enrichment analysis of genes co-expressed with GLUT1 in PAAD. (A) Genes were co-expressed with GLUT1 in the TCGA pancreatic adenocarcinoma (TCGA-PAAD) dataset by Pearson test. (B) The heatmap of the top 50 genes positively correlated with GLUT1 in the TCGA-PAAD dataset. (C) The heatmap of the top 50 genes negatively correlated with GLUT1 in the TCGA-PAAD dataset. (D) Enrichment analysis of GO terms and KEGG pathways for genes differently co-expressed with GLUT1. The top 10 enriched GO-BP terms. (E) The top 10 enriched GO-CC terms. (F) The top 10 enriched GO-MF terms. (G) The top 10 enriched KEGG pathways. (H) Enrichment plot of focal adhesion according to GESA. (I) PPI network centered on GLUT1 by STRING database.

LinkedOmics database was utilized for screening the genes which co-expressed with GLUT1 in PAAD. Among all genes which co-expressed with GLUT1, 4156 genes were synchronously upregulated with GLUT1, whereas 6345 genes were synchronously downregulated with GLUT1 (Figure 8A). In Figures 8B and 8C, heatmaps illustrated the top 50 significantly differentially expressed genes that were upregulated and downregulated synchronously, and details of the data were presented in Supplementary Table 1 and Supplementary Table 2. GO and KEGG enrichment analyses of the altered genes mentioned above were carried out to detect their biological functions. The results of GO consisted of biological process (GO-BP), cellular component (GO-CC), molecular functional (GO-MF), and the enrichment analyses were shown in the form of bubble plots (Figure 8D-G). The bubble plots listed the top 10 enriched terms of GO-BP, GO-CC, GO-MF respectively, and the top 10 enriched KEGG pathways. The relevant terms were cell junction organization, contractile fiber part, and identical protein binding, and the relevant pathways were metabolic pathways, pathways in cancer, and focal adhesion. The enrichment plot of focal adhesion was shown in Figure 8H. The PPI network centered on GLUT1 was analyzed through the STRING tool (Figure 8I). All the above results revealed that GLUT1 participated in the tumor progression of PAAD.

### GLUT1 expression and function in PAAD tissues and cell lines

**Figure 9:**
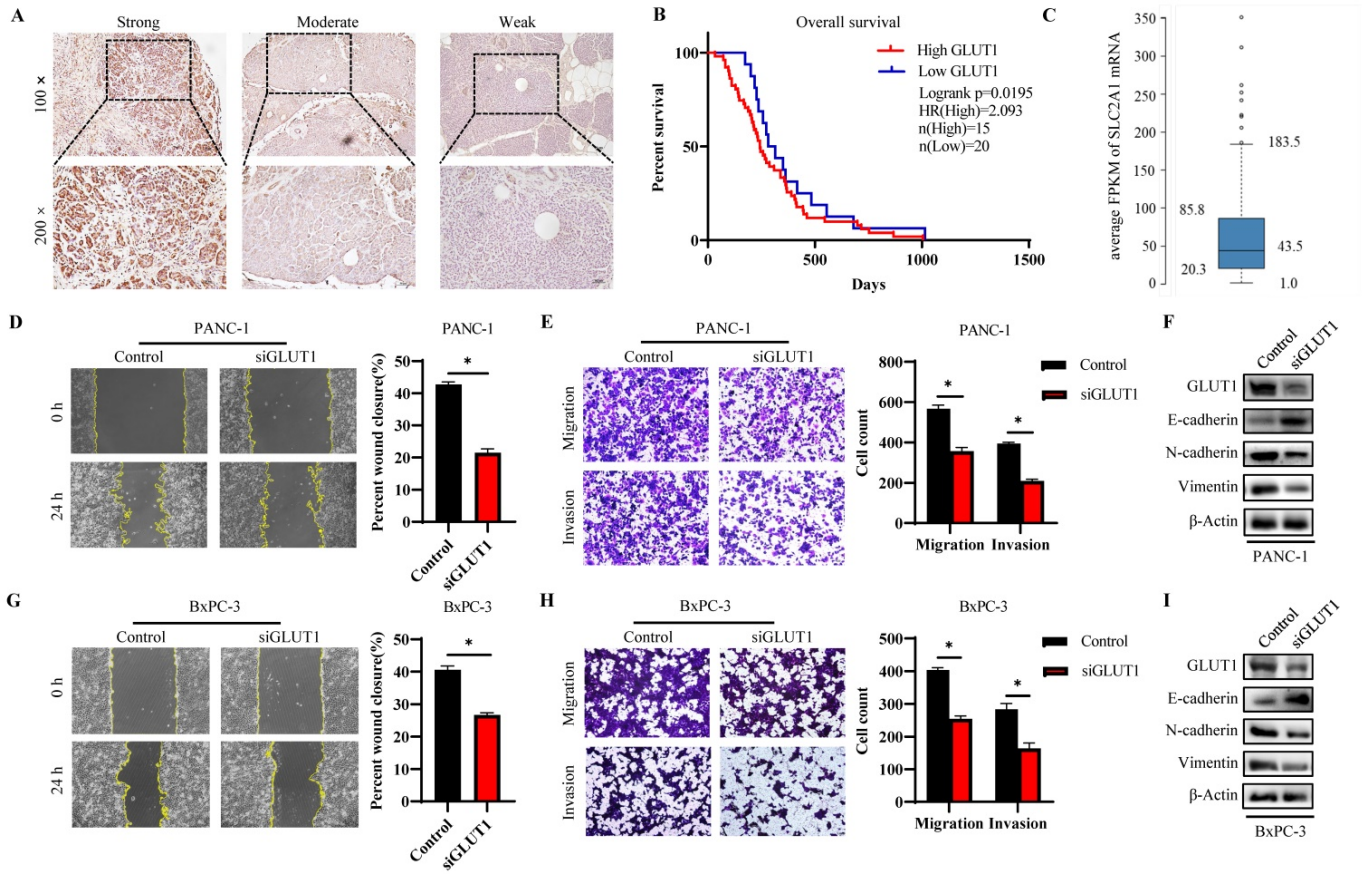
GLUT1 expression and function in PAAD tissues and cell lines. (A) Representative images of different IHC staining intensities of GLUT1 in PAAD tissues. Scale bars represent 50 μm (×10 magnification images) and 50 μm (×20 magnification images). (B) Kaplan-Meier survival curve related to GLUT1 expression. (C) Boxplots of GLUT1 mRNA expression in FPKM in PAAD sample from the Human Protein Atlas. (D) Transwell assays of PANC-1 cells and GLUT1 knockdown PANC-1 cells. Representative images of the scratch assay were acquired at 0 h and 24 h after making scratches. (E) The covered scratched area by PANC-1 cells was calculated in percent. (F) Western blot analysis of EMT-related proteins (E-cadherin and N-cadherin) of PANC-1 cells and GLUT1 knockdown PANC-1 cells. (G) Transwell assays of BxPC-3 cells and GLUT1 knockdown BxPC-3 cells. Representative images of the scratch assay were acquired at 0 h and 24 h after making scratches. (H) The covered scratched area by BxPC-3 cells was calculated in percent. (I) Western blot analysis of EMT-related proteins of BxPC-3 cells and GLUT1 knockdown BxPC-3 cells.

The GLUT1 expression level in human pancreatic tumor tissues was verified by IHC analysis to further validate the findings of highly expressed GLUT1 in PAAD in the aforementioned pan-cancer analysis. Representative images with strong, moderate, and weak intensities of GLUT1 expression were presented in Figure 9A. Strong positive or moderately positive results were more frequently in tumor tissues, and weak positive or negative results were more frequently in adjacent tissues. Kaplan-Meier survival analysis based on GLUT1 expression levels, which were separated into two groups based on IHC staining: high GLUT1 expression and low GLUT1 expression. Patients with high GLUT1 expression levels tended to have shorter survival times (Figure 9B). We also acquired the GLUT1 mRNA expression from the Human Protein Atlas (Human Protein Atlas proteinatlas.org)^29^, and the GLUT1 mRNA was highly expressed in pancreatic cancer tissues with an average abundance of expression of 43.5 FPKM (fragments per kilobase of transcript per million fragments mapped) and ranging from 20.3 FPKM to 85.8 FPKM (Figure 9C). In light of the enrichment analysis results, we speculated that cell metastatic ability is correlated with GLUT1 expression. PANC-1 cells and BxPC-3 cells were transfected with siRNA respectively, and western blot analysis was used to detect the knockdown efficiency of GLUT1 (Figure 9F and Figure 9I). Transwell assay and scratch assay were used to explore the effects of different treatments on the migration and invasion of pancreatic cancer cells (Figure 9D-E and Figure 9G-H). The experimental results demonstrated that GLUT1 knockdown significantly attenuated the migration and invasion abilities of pancreatic cancer cells. GLUT1 knockdown also decreased the expression levels of Epithelial-mesenchymal transition (EMT) related proteins as a result of the downregulation of the epithelial protein E-cadherin and the upregulation of the mesenchymal protein N-cadherin and Vimentin (Figure 9F and Figure 9I).

## Discussion

Patients with PAAD have a worse prognosis for the poor treatment response and ease of recurrence and metastasis 30, 31. The pan-cancer analysis will facilitate the discovery of effective diagnostic factors. Our study found abnormally highly expressed GLUT1 in multiple human cancer types using the GEPIA database, which correlated with worse prognosis in PAAD. GLUT1 had been proven to be an indicator of aggressive biological function and poor prognosis in various human cancer types 32-35. Further research is needed to determine the specific regulatory mechanisms of this gene and its role in the progression of PAAD.

Our study focused on miRNAs, which act as the upstream regulatory factors of GLUT1. Previous studies demonstrated that ncRNAs have a potential role in regulating the expression of genes by influencing their stability 36, 37. According to the bioinformatics analysis based on the StarBase database and TargetScan database, several miRNAs were found to be the possible upstream miRNAs regulating GLUT1. MiR-140-5p was found to have the highest probability of directly targeting GLUT1 by differential expression analysis and correlation analysis. Survival analysis demonstrated that patients with a low expression level of miR-140-5p tended to own a worse prognosis.

Increasing evidence favors the ceRNA hypothesis in various cancer types 38-41. LncRNAs act as ceRNAs that interact competitively with shared miRNAs and are involved in regulating the target genes of miRNAs 42-44. Using the online tools, we screened the upstream lncRNAs targeting miR-140-5p and finally found that CASC19 had the highest odds of being the upstream ncRNA of miR-140-5p in PAAD. Additionally, CASC19 overexpression indicated poor OS and DFS in PAAD patients. As previously proven, CASC19 was regarded as a potential oncogene through the miR-140-5p/GLUT1 axis in PAAD. Finally, we established a regulatory network associated with prognosis and the immune system and provided a possible lncRNA-miRNA-mRNA axis in PAAD.

Tumor immune cell infiltration takes part in cancer progression and prognosis 45, 46. The infiltrating degree of immune cells potentially had an impact on the efficacy of immunotherapy in multiple human cancer types, particularly in PAAD 47, 48. According to our results, the copy number variations of GLUT1 caused considerable alterations in the infiltration of the multiple immune cell populations. Furthermore, its expression level was remarkably correlated with the infiltration levels as well as the corresponding biomarkers of immune cells mentioned above. And the immunotherapy effect was largely affected by the expression of immune checkpoints 49, which contributes to various prognosis 50, 51. In our study, GLUT1 overexpression significantly correlated with low CD8+ T-cell and CD4+ T-cell infiltration, and its expression was positively related to PD-L1 and negatively related to PD-1 in PAAD. All the results together revealed that GLUT1 might participate in the immune microenvironment and influence the effect of immunotherapy.

We explored the co-expressed genes with GLUT1 and the co-expressed genes were involved in cell junction organization, contractile fiber part, and identical protein binding. KEGG pathway analysis pointed out that genes co-expressed with GLUT1 were mostly enriched in metabolic pathways, pathways in cancer, and focal adhesion. PPI analysis revealed that GLUT1 correlated with GIPC1, SERPINH1, HIF1A, TP53, and LDHA. As discussed above, GLUT1 was highly expressed, and we also validated the expression level of GLUT1 by IHC staining using clinical specimens. The GLUT1 expression level was closely correlated with clinical characteristics, including tumor size, the degree of tumor differentiation, and the status of lymph node metastasis. All the above results indicated that GLUT1 participated in tumor initiation and progression of PAAD. Previous studies have found that GLUT1 is essential for glucose uptake by cancer cells, and enhanced glucose uptake maintains the malignant proliferation of cancer cells 52. According to enrichment analysis results, in addition to the metabolic pathway, GLUT1 co-expressed genes were also mainly related to focal adhesion. Focal adhesion acted as a signal transduction center and participates in the regulation of growth and metastasis of cancer cells 53. After analyzing the PPI network of genes related to GLUT1, we found that GIPC1 54, 55, and SERPINH1 56, 57 were related to the mechanism of tumor metastasis, including in pancreatic tumors. To evaluate the role of GLUT1 in regulating PAAD cells metastasis, GLUT1 was knocked down in cells and assessed the metastatic abilities. Our initial analysis has revealed that GLUT1 may act as a potential factor to promote the metastasis of cancer cells.

## Conclusions

To sum up, our study utilized online databases which contain the data of the TCGA and GTEx databases to perform expression and prognostic association analysis across pan-cancer and discovered that GLUT1 was overexpressed in multiple human cancer types, including PAAD. Survival analysis revealed that patients with high GLUT1 expression tended to have a shorter survival time than those with low expression. Then we predicted the upstream miRNAs of GLUT1 mRNA and lncRNAs of candidate miRNA using prediction databases, and established ncRNAs regulatory network based on miR-140-5p/GLUT1 mRNA and CASC19/miR-140-5p interactions. Results revealed that GLUT1 might impact tumor immune microenvironment and metastasis. Furthermore, we assessed the expression level of GLUT1 in clinical pancreatic cancer samples, and its positive rate correlated with survival and clinical characteristics, including tumor size, the degree of tumor differentiation, and the status of lymph node metastasis. We initially confirmed the promoting role of GLUT1 in tumor metastasis by functional experiments. These results suggested that GLUT1 had the potential to serve as an indicator of poor prognosis, as well as influencing the tumor immune microenvironment and promoting tumor metastasis in PAAD. However, further research is still needed to discover the specific mechanism underlying GLUT1 in the future.

## Supplementary Material

Supplementary tables.

## Figures and Tables

**Table 1:** Correlation analysis between lncRNA and GLUT1 mRNA in PAAD.

lncRNA	mRNA	*R*-value	*P*-Value
LINC01816	SLC2A1	-0.223	2.72E-03**
FGD5-AS1	SLC2A1	0.165	2.76E-02*
NR2F1-AS1	SLC2A1	0.279	1.60E-04***
HCP5	SLC2A1	0.234	1.65E-03**
MSC-AS1	SLC2A1	0.149	4.74E-02*
CASC19	SLC2A1	0.365	5.41E-07***
PVT1	SLC2A1	0.481	1.03E-11***
PRKCQ-AS1	SLC2A1	-0.273	2.23E-04***
H19	SLC2A1	0.198	7.97E-03**
SBF2-AS1	SLC2A1	0.24	1.28E-03**
SNHG1	SLC2A1	0.213	4.27E-03**
CD27-AS1	SLC2A1	0.188	1.18E-02*
LINC01588	SLC2A1	0.419	6.02E-09***
OIP5-AS1	SLC2A1	0.196	8.88E-03**
SNHG20	SLC2A1	-0.176	1.88E-02*
LINC00667	SLC2A1	-0.241	1.19E-03**
LINC01534	SLC2A1	-0.3	4.82E-05***
SDCBP2-AS1	SLC2A1	-0.341	3.18E-06***
TUG1	SLC2A1	0.275	2.07E-04***
LINC00894	SLC2A1	0.175	2.72E-03*

*P<0.05; **P<0.01; ***P<0.001.

**Table 2:** Correlation analysis between SLC2A1 and biomarkers of immune cells in PAAD

Immune cell	Biomarker	*R*-value	*P*-value
B cell	CD19	-0.143	6.27E-02
	CD20	-0.207	6.54E-03**
	CD70	0.27	3.54E-04***
	CD79A	-0.173	2.35E-02*
CD+4 Tcell	CD4	-0.092	2.34E-01
CD+8 Tcell	CD8A	-0.283	1.72E-04***
	CD8B	-0.213	5.14E-03**
	CD25	0.094	2.20E-01
Macrophage	CD68	0.205	7.12E-03**
	CD11B	0.1	1.95E-01
M1 Macrophage	NOS2	-0.033	6.64E-01
	IRF5	0.068	3.74E-01
	COX2	0.491	9.43E-12***
M2 Macrophage	CD163	-0.078	3.09E-01
	CD206	-0.061	4.26E-01
	VSIG4	-0.039	6.09E-01
	MS4A4A	-0.144	6.02E-02
Neutropil	CD16	0.093	2.25E-01
	CD55	0.573	2.52E-16***
	CEACAM8	0.059	4.41E-01
	ITGAM	0.1	1.95E-01
	CCR7	0.189	1.31E-02*
Dendritic cell	CD141	0.041	5.92E-01
	HLA-DPB1	-0.23	2.43E-03**
	HLA-DQB1	-0.104	1.75E-01
	HLA-DRA	-0.127	9.91E-02
	HLA-DPA1	-0.176	2.17E-02*
	CD1C	-0.184	1.58E-02*
	BDCA-4	0.049	5.24E-01
	CD11C	0.023	7.63E-01

*P<0.05; **P<0.01; ***P<0.001.

**Table 3:** Clinicopathologic characteristics of the patients with PAAD.

Characteristics	High GLUT1 expression	Low GLUT1 expression	χ^2^	*P*-Value
	n (%)	n (%)		
Gender			0.285	0.594
Male	32(74.42%)	11(25.58%)		
Female	20(83.33%)	4(16.67%)		
Age			0.082	0.774
≥50	42(77.78%)	12(22.22%)		
<50	9(69.23%)	4(30.77%)		
Tumor size			4.411	0.036*
≥3cm	46(80.70%)	11(19.30%)		
<3cm	5(50.00%)	5(50.00%)		
T stage			0.225	0.635
T1-T2	8(66.67%)	4(33.33%)		
T3-T4	43(78.18%)	12(21.82%)		
N-stage			5.553	0.018**
N0	18(62.07%)	11(37.93%)		
N1	33(86.84%)	5(13.16%)		
M-stage			1.352	0.245
M0	39(72.22%)	15(27.78%)		
M1	12(92.31%)	1(7.69%)		
Pathologic stage			1.055	0.304
I-II	36(72.00%)	14(28.00%)		
III-IV	15(88.24%)	2(11.76%)		
Differentiation			6.013	0.049*
Well	13(59.09%)	9(40.91%)		
Moderately	19(79.17%)	5(20.83%)		
Poorly	19(90.48%)	2(9.52%)		

*P<0.05; **P<0.01.

## Abbreviations

PAAD: pancreatic adenocarcinoma
GLUT1: glucose transporter 1
SLC2A1: Solute carrier family 2 member 1
ncRNA: non-coding RNA
ceRNA: competitive endogenous RNA
TCGA: The Cancer Genome Atlas
GTEx: The Genotype-Tissue Expression
GO: Gene Ontology
KEGG: Kyoto Encyclopedia of Genes and Genomes
GSEA: Gene Set Enrichment Analysis
PPI: protein-protein interaction
CTLA-4: cytotoxic T-lymphocyte-antigen-4
PD-1: programmed cell death protein 1
PD-L1: programmed cell death ligand 1
siRNA: small interfering RNA
OS: overall survival
DFS: disease-free survival
EMT: epithelial-mesenchymal transition
PVDF: polyvinylidene difluoride
FPKM: fragments per kilobase of transcript per million fragments mapped
TPM: transcripts per million
