# Supplementary material for: GLUT1 Regulates the Tumor Immune Microenvironment and Promotes Tumor Metastasis in Pancreatic Adenocarcinoma via ncRNA-mediated Network
Source: J Cancer. 2022 May 13;13(8):2540–58. doi: 10.7150/jca.72161 (PMC9174867; doi:10.7150/jca.72161)
Supplement: Supplementary file 1 — Supplementary tables. [file jcav13p2540s1.pdf]

Supplementary tables

Table S1. The top 50 significantly differentially expressed genes that were upregulated synchronously with GLUT1.

Gene	Statistic	P-value	FDR
SLC2A1	1	1.00E-33	1.00E-29
EPHA2	0.748131	3.57E-33	3.53E-29
CDCP1	0.745665	7.45E-33	3.72E-29
CDH3	0.745635	7.52E-33	3.72E-29
AIM1L	0.722083	5.69E-30	2.25E-26
ITGA3	0.718694	1.40E-29	4.60E-26
ACTN4	0.716867	2.25E-29	6.36E-26
ADM	0.716266	2.64E-29	6.51E-26
SEMA4B	0.715105	3.56E-29	7.83E-26
TMEM189	0.708851	1.76E-28	3.48E-25
SLC16A3	0.702936	7.68E-28	1.38E-24
LDHA	0.698923	2.04E-27	3.37E-24
COX6B2	0.695268	4.92E-27	7.48E-24
PLEC	0.691571	1.18E-26	1.66E-23
MALL	0.691223	1.28E-26	1.69E-23
TINAGL1	0.689543	1.89E-26	2.34E-23
ALDOA	0.686201	4.10E-26	4.77E-23
TFAP2A	0.684101	6.63E-26	7.28E-23
FAM83A	0.682942	8.62E-26	8.85E-23
PPARG	0.682407	9.73E-26	9.17E-23
SERPINB5	0.680214	1.60E-25	1.43E-22
PPP1R3G	0.679081	2.06E-25	1.77E-22
ASAP2	0.678352	2.42E-25	1.99E-22
HK2	0.676864	3.36E-25	2.66E-22
SDC4	0.673254	7.44E-25	5.66E-22
S100A10	0.671482	1.09E-24	8.01E-22
ERO1L	0.670838	1.26E-24	8.88E-22
LRRC8A	0.670577	1.33E-24	9.07E-22
SCEL	0.668295	2.17E-24	1.43E-21
LAMC2	0.667811	2.41E-24	1.52E-21
BHLHE40	0.667689	2.47E-24	1.52E-21
PLEKHN1	0.66758	2.53E-24	1.52E-21
GNA15	0.665326	4.08E-24	2.38E-21
JUP	0.663902	5.52E-24	3.12E-21
FLNB	0.659368	1.42E-23	7.80E-21
PCDH1	0.658728	1.62E-23	8.66E-21
MBOAT2	0.654831	3.60E-23	1.83E-20

FOXL1	0.654082	4.19E-23	2.07E-20
PLEK2	0.651752	6.71E-23	3.23E-20
C8orf73	0.650162	9.22E-23	4.34E-20
SERINC2	0.649799	9.92E-23	4.46E-20
GJB3	0.649326	1.09E-22	4.79E-20
PKM2	0.645458	2.34E-22	9.45E-20
ELOVL1	0.644548	2.80E-22	1.11E-19
RHOC	0.644118	3.04E-22	1.18E-19
INPP4B	0.64346	3.46E-22	1.32E-19
LAMB3	0.642017	4.58E-22	1.71E-19
LOC100131726	0.640185	6.52E-22	2.39E-19
MET	0.639549	7.37E-22	2.60E-19
CGB7	0.638732	8.62E-22	2.99E-19

Table S2. The top 50 significantly differentially expressed genes that were downregulated synchronously with GLUT1.

Gene	Statistic	P-value	FDR
FBXO9	-0.68278	8.95E-26	8.85E-23
ELAC1	-0.65768	2.01E-23	1.05E-20
RWDD2A	-0.64992	9.67E-23	4.45E-20
ZFP3	-0.64916	1.13E-22	4.84E-20
SESN1	-0.64631	1.98E-22	8.32E-20
RABEP1	-0.64592	2.14E-22	8.81E-20
ACACB	-0.64006	6.68E-22	2.40E-19
GSTA4	-0.6367	1.27E-21	4.19E-19
TTLL11	-0.6329	2.60E-21	7.79E-19
EPM2A	-0.63182	3.18E-21	9.26E-19
HDHD2	-0.63173	3.24E-21	9.27E-19
LYRM2	-0.6296	4.81E-21	1.32E-18
ACSL6	-0.62717	7.52E-21	1.98E-18
ACAT1	-0.62551	1.02E-20	2.47E-18
ZNF181	-0.62548	1.02E-20	2.47E-18
ZNF596	-0.62408	1.32E-20	3.11E-18
EPHX2	-0.62373	1.41E-20	3.28E-18
SEC11C	-0.62251	1.76E-20	3.95E-18
B3GNT1	-0.62166	2.05E-20	4.36E-18
GKAP1	-0.61769	4.16E-20	8.07E-18
MOCS2	-0.61698	4.72E-20	8.97E-18
EML5	-0.61128	1.28E-19	2.18E-17
C22orf39	-0.60956	1.72E-19	2.89E-17
NCOA5	-0.60813	2.21E-19	3.58E-17
LOC400657	-0.60787	2.30E-19	3.68E-17

CYB5D2	-0.60722	2.58E-19	4.04E-17
SRR	-0.60468	3.97E-19	6.04E-17
FAM184A	-0.59768	1.28E-18	1.76E-16
RUNDC3B	-0.59735	1.35E-18	1.83E-16
ZNF491	-0.5972	1.38E-18	1.86E-16
C20orf132	-0.59468	2.10E-18	2.69E-16
C9orf130	-0.5938	2.42E-18	3.05E-16
ZNF658	-0.59332	2.62E-18	3.24E-16
PARK2	-0.59201	3.24E-18	3.84E-16
TATDN3	-0.59116	3.71E-18	4.34E-16
RFXAP	-0.58905	5.22E-18	5.96E-16
METTL7A	-0.58626	8.15E-18	8.85E-16
ZNF271	-0.58607	8.39E-18	9.07E-16
PDCD4	-0.58575	8.83E-18	9.48E-16
ASB8	-0.58515	9.71E-18	1.03E-15
ATP5A1	-0.58483	1.02E-17	1.07E-15
SOCS2	-0.58353	1.25E-17	1.29E-15
JMJD5	-0.583	1.36E-17	1.38E-15
C6orf89	-0.57957	2.33E-17	2.24E-15
SCML2	-0.57904	2.53E-17	2.39E-15
C20orf160	-0.57823	2.87E-17	2.67E-15
TCEAL1	-0.57811	2.92E-17	2.71E-15
TSPAN7	-0.57808	2.93E-17	2.71E-15
SNTG2	-0.57753	3.19E-17	2.91E-15
PLK1S1	-0.57741	3.25E-17	2.95E-15
